# Causes of death among cancer patients in the era of cancer survivorship in Korea: Attention to the suicide and cardiovascular mortality

**DOI:** 10.1002/cam4.2813

**Published:** 2020-01-20

**Authors:** Chang‐Mo Oh, Dahhay Lee, Hyun‐Joo Kong, Seokmin Lee, Young‐Joo Won, Kyu‐Won Jung, Hyunsoon Cho

**Affiliations:** ^1^ Department of Preventive Medicine School of Medicine Kyung Hee University Seoul Republic of Korea; ^2^ Department of Cancer Control and Population Health Graduate School of Cancer Science and Policy National Cancer Center Goyang Republic of Korea; ^3^ Division of Cancer Registration and Surveillance National Cancer Control Institute National Cancer Center Goyang Republic of Korea; ^4^ Vital Statistics Division Statistics Korea Daejeon Republic of Korea

**Keywords:** cancer survivorship, cardiovascular diseases, cause of death, neoplasms, noncancer mortality, suicide

## Abstract

**Background:**

Improved cancer survival is expected to increase noncancer deaths; however, detailed causes of death have rarely been discussed. Changing landscapes in mortality patterns and noncancer mortality risks in patients with cancer require evaluation.

**Methods:**

We identified cancer and noncancer‐related causes of death using data from the 2000‐2016 national cancer registry in Korea (n = 2 707 520), and we characterized the leading causes of death and proportionate mortalities over time. Risks of noncancer deaths relative to the general population were estimated using standardized mortality ratios (SMRs).

**Results:**

Of 1 105 607 identified deaths, 87% were due to the primary cancer. Proportionate mortalities of primary cancer among overall deaths remained high in patients with liver (86%) and lung (70%) cancers and in female patients with breast cancer (77%), even 5 to 10 years following diagnosis, whereas proportionate mortalities reduced to ≤50% in patients with stomach (men, 39%; women, 48%), prostate (47%), and female thyroid (27%) cancers. Despite the predominance of index cancer deaths, the proportion of noncancer deaths among all deaths increased over time. There was a 20‐fold increase in cardiovascular disease deaths among patients with cancer from 2000 to 2016, and the risk of suicide among patients with cancer was higher than that among the general population (SMR: 1.68 [95% confidence interval (CI): 1.63‐1.74] in men, SMR: 1.42 [95% CI: 1.33‐1.55] in women).

**Conclusions:**

Deaths from primary cancer remain a major concern; however, follow‐up is required for both cancer and noncancer‐related health issues in cancer survivors, especially concerning suicide and cardiovascular deaths.

## Graphical abstract

Death from primary cancer is still the major concern, but noncancer deaths are expected to become an important issue. Higher risks of suicide death and increasing number of cardiovascular disease deaths in cancer patients highlight the need for preventing suicide and cardiovascular disease in cancer patients.

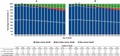

## 1 INTRODUCTION

Cancer is the leading cause of death worldwide, including in South Korea.^1^ According to the GLOBOCAN, 18.1 million cancer cases were newly diagnosed and 9.6 million people died from cancer in 2018.^2^ Although the burden of cancer has steadily increased, survival for patients with cancer has also improved in developed countries in response to better cancer prevention, screening, and treatment strategies.^3^ As patients are living longer, mortality from noncancer causes and secondary cancer‐related deaths are increasing, and in‐depth research on this matter has become more urgent. In South Korea, cancer survival has improved rapidly in recent decades, as many patients with cancer survive more than 5 years after diagnosis (5‐year relative survival rate: 70.6% in 2016), and, to date, the estimated number of cancer survivors exceeds 1.7 million.^1^ Issues related to acute cancer care are transitioning to long‐term cancer survivorship.

The landscape of mortality among patients with cancer is also changing. Zaorsky et al^4^ reported that the burden of noncancer deaths among patients with specific cancers has recently increased more than the risk of cancer deaths in the United States. One study has reported that women with endometrial cancer were more likely to die from cardiovascular disease than from the endometrial cancer,^5^ and men diagnosed with prostate cancer have a higher risk of dying from other diseases, rather than prostate cancer itself, based on Surveillance, Epidemiology, and End Results (SEER) data.^6^ However, primary cancer has been reported to be the leading cause of death among patients with head and neck cancer^7^ or diffuse large B‐cell lymphoma.^8^

Although annual reports on cancer statistics often include survival and mortality data,^1^, ^9^ nationwide comprehensive reviews regarding causes of death among patients with cancer are limited.^4^ For each type of cancer, some studies have evaluated the main cause of cancer death in the clinical setting.^5^, ^6^, ^7^, ^8^ However, very few studies, for example, one study undertaken in the United States recently, have conducted a comprehensive population‐based cause of death assessment of overall patients with cancer.^4^ Moreover, in most developing countries, reliable cause of death data are often unavailable or population‐based cancer registry data are not linked to mortality. In South Korea, causes of death statistics in the general population have routinely been reported as official statistics by Statistics Korea^10^; however, a nationwide review of causes of death among all Korean patients with cancer remains to be conducted. Data derived from a nationwide population‐based Korean cancer registry linking mortality with cause of death statistics provide an opportunity to investigate this matter.

This paper aimed to characterize the causes of death and investigate the nationwide mortality patterns in South Korean patients with cancer in relation to long‐term cancer survivorship. We identified the leading causes of death, the proportional mortality of cancer, and the risks of noncancer deaths in patients with solid tumors most common in the South Korean population (stomach, colorectal, liver, lung, prostate, female breast, and thyroid cancers).

## 2 MATERIALS AND METHODS

### 2.1 Study population

We obtained data from a population‐based cancer registry, the Korea Central Cancer Registry (KCCR), which is linked to mortality statistics data. This retrospective cohort database included patients diagnosed with malignant primary tumors between 2000 and 2016, who had been followed until 31 December 31, 2016. The KCCR is a nationally representative, population‐based cancer registry covering >99% of patients diagnosed with cancer in South Korea and contains nationwide cancer incidence and survival data from 1999 onward. Furthermore, the registry is linked to cause of death statistics provided by Statistics Korea.^1^, ^11^ Statistics Korea collects vital status and cause of death data from death certificates and classifies the causes of death according to the International Statistical Classification of Diseases and Related Health Problems, 10th revision (ICD‐10), as recommended by the World Health Organization (WHO).^10^, ^12^ The study protocol was approved by the institutional review board of the National Cancer Center (NCC2016‐0041).

### 2.2 Cause of death classification

We used underlying causes of death provided by the National Statistical Office in South Korea as the primary end point in this study. Compiling statistics on the causes of death is undertaken, in accordance with WHO 2010 guidelines, by the National Statistical Office in South Korea. The underlying causes of death were coded according to ICD‐10 codes.^12^ Cancer death refers to a death due to a diagnosed cancer. In this study, we defined death due to the first primary cancer diagnosed as an index cancer death,^4^ and death due to a diagnosed cancer other than the first primary cancer as a nonindex cancer death.^4^ Noncancer death was defined as a death attributed to causes other than cancer. We applied the SEER cause of death classification algorithm to reclassify cause of death (cancer vs noncancer) in patients with cancer.^13^ This algorithm was developed and extensively validated in the United States National Cancer Center Institute's SEER program to improve the accuracy of underlying cause of death information in patients with cancer through correcting possible misclassifications.^13^, ^14^ To determine the cause of death rankings among patients with cancer relative to a general population, we used groupings in a list of 56 causes of death. The list was selected from a set of 80 causes of death recommended by the WHO and is officially used by Statistics Korea to determine the cause of death rankings in the South Korean general population.^10^, ^11^

### 2.3 Statistical analysis

Descriptive statistics on demographics, tumor characteristics, and proportions of death according to causes (index cancer death, nonindex cancer death, and noncancer death) were calculated for all patients with cancer for all age groups and according to cancer type; the latter being restricted to cancers common in the South Korean population. We estimated proportionate mortality, defined as the number of deaths from a specific cause divided by the number of total deaths, to describe the distributions of mortality according to cause of death and to illustrate changes in the cause of death distribution in patients with cancer according to time since diagnosis (<5, 5‐10, and ≥10 years).^15^ We calculated rankings for causes of death and the leading causes of death in patients with cancer and compared the results to the causes of death in the Korean general population. All age groups were included to calculate proportional mortality or calculate rankings in terms of the cause of death.

We estimated standardized mortality ratios (SMRs) and 95% confidence intervals (CIs) to compare mortality risks of noncancer diseases in patients with cancer relative to the Korean general population in the 5‐year period from 2012 to 2016. We first calculated age‐specific mortality rates for the major causes of death (using the list of 56 causes of death previously noted) in the general population using 5‐year age groups (from ≥20 years to <85 years old), and estimated the expected number of noncancer causes of death in patients with cancer through applying the estimated age‐specific mortality rates to the corresponding cancer patient population. We then calculated the SMR for a specific noncancer cause of death in patients with cancer relative to the general population as the observed number of deaths divided by the expected number of deaths in patients with cancer. The analyses were stratified according to sex. The 95% CI for the SMR was calculated using the method described by Kahn and Sempos.^16^ Absolute excess risk (AER) was also calculated as the difference between the expected and the observed death rate per 10 000 person‐years. When calculating SMR and AER, children and adolescents aged <20 and elderly people aged ≥85 years old were excluded.^17^, ^18^ The statistical analyses were performed using SAS 9.4 (SAS Institute) and Excel 2013 (Microsoft).

## 3 RESULTS

### 3.1 Characteristics of death among patients with cancer

Of 2 707 520 patients with cancer diagnosed between 2000 and 2016, 1 105 607 patients (40.8%) had died at the end of 2016; of which 87.2% of deaths were due to the primary cancer, 3.0% of deaths were due to nonindex cancer, and 9.8% of deaths were due to noncancer‐related causes (Table 1). The mean age at cancer diagnosis was 59.3 years and patient age varied according to cancer types. Breast cancer was diagnosed at an early age (mean age, 50.6 years), whereas prostate cancer was diagnosed at an older age (mean age, 69.9 years).

**Table 1:** Characteristics and number of death in cancer patients diagnosed between 2000 and 2016 in Korea

Characteristics	All sites (N = 2 707 520)		Stomach (n = 434 956)		Colorectal (n = 334 320)		Liver (n = 231 884)		Lung (n = 279 190)		Breast^a^ (n = 213 034)		Thyroid (n = 369 824)		Prostate (n = 96 917)	
Age	Mean	SD	Mean	SD	Mean	SD	Mean	SD	Mean	SD	Mean	SD	Mean	SD	Mean	SD
Age at diagnosis	59.3	15.1	62.1	12.9	63.3	12.7	60.8	12.3	67.5	11.1	50.6	11.4	47.3	12.3	69.9	8.5
Age at death	67.6	13.6	68.1	13.4	70.4	12.8	63.2	12.4	69.9	10.6	58.2	14.6	69.3	13.1	77.5	8.6

	Count	(%^b^)	Count	(%^b^)	Count	(%^b^)	Count	(%^b^)	Count	(%^b^)	Count	(%^b^)	Count	(%^b^)	Count	(%^b^)
Sex																
Men	1 410 800	(52.1)	291 488	(67.0)	197 965	(59.2)	174 931	(75.4)	200 563	(71.8)			64 729	(17.5)	96 917	(100.0)
Women	1 296 720	(47.9)	143 468	(33.0)	136 355	(40.8)	56 953	(24.6)	78 627	(28.2)	213 034	(100.0)	305 095	(82.5)		
Alive	1 601 913	(59.2)	256 536	(59.0)	216 335	(64.7)	59 126	(25.5)	63 940	(22.9)	186 085	(87.3)	360 508	(97.5)	70 620	(72.9)
Death	1 105 607	(40.8)	178 420	(41.0)	117 985	(35.3)	172 758	(74.5)	215 250	(77.1)	26 949	(12.7)	9 316	(2.5)	26 297	(27.1)

	Count	(%^c^)	Count	(%^c^)	Count	(%^c^)	Count	(%^c^)	Count	(%^a^, ^c^)	Count	(%^c^)	Count	(%^c^)	Count	(%^c^)
Death																
Cause of death																
Index cancer	964 192	(87.2)	146 211	(81.9)	96 885	(82.1)	163 585	(94.7)	204 463	(95.0)	22 508	(83.5)	4 288	(46.0)	16 317	(62.0)
Nonindex cancer	32 903	(3.0)	6 618	(3.7)	4 277	(3.6)	2 754	(1.6)	1 512	(0.7)	1 278	(4.7)	1 795	(19.3)	2 257	(8.6)
Noncancer	108 512	(9.8)	25 591	(14.3)	16 823	(14.3)	6 419	(3.7)	9 275	(4.3)	3 163	(11.7)	3 233	(34.7)	7 723	(29.4)
Time to death since diagnoses																
0‐5 y	983 771	(89.0)	153 067	(85.8)	99 301	(84.2)	160 871	(93.1)	206 571	(96.0)	18 306	(67.9)	5 894	(63.3)	18 744	(71.3)
5‐10 y	94 191	(8.5)	18 420	(10.3)	14 438	(12.2)	10 268	(5.9)	7 037	(3.3)	6 581	(24.4)	2 518	(27.0)	6 135	(23.3)
10+ y	27 645	(2.5)	6 933	(3.9)	4 246	(3.6)	1 619	(0.9)	1 642	(0.8)	2 062	(7.7)	904	(9.7)	1 418	(5.4)

Note

Cancer patients were diagnosed from 2000 to 2016 and vital status was followed until 31 December 2016.

Abbreviation: SD, standard deviation.

a Female only.

b Percentage of total number of cancer patients, which was calculated as the number of specific patient characteristic divided by the total number of patients.

c Percentage of total number of deaths, which was calculated as the number of specific patient characteristic divided by the total number of death.

The highest frequencies of primary cancer‐related deaths were observed among patients with lung or liver cancer (204 463 deaths and 163 585 deaths, respectively), and approximately 4% of total deaths in these groups were noncancer‐related. In contrast, 34.7% of deaths in patients with thyroid cancer and 29.4% of deaths in patients with prostate cancer were noncancer‐related. More than 10% of the total deaths among patients with stomach, colorectal, and female breast cancer were also due to noncancer causes.

### 3.2 Changes in mortality patterns: number of deaths and proportionate mortality according to cause of death

**Figure 1:**
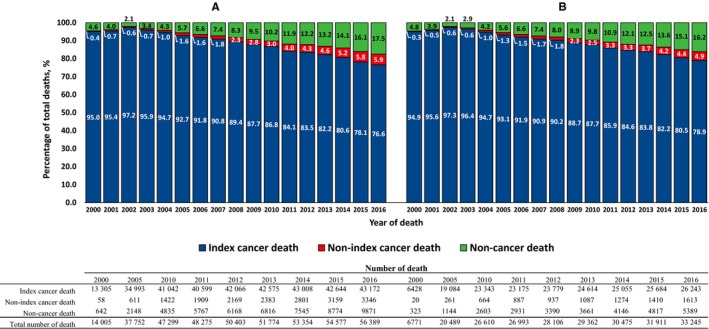
Proportion of cause of death among (A) male and (B) female cancer patients with primary cancer diagnosed between 2000 and 2016

**Figure 2:**
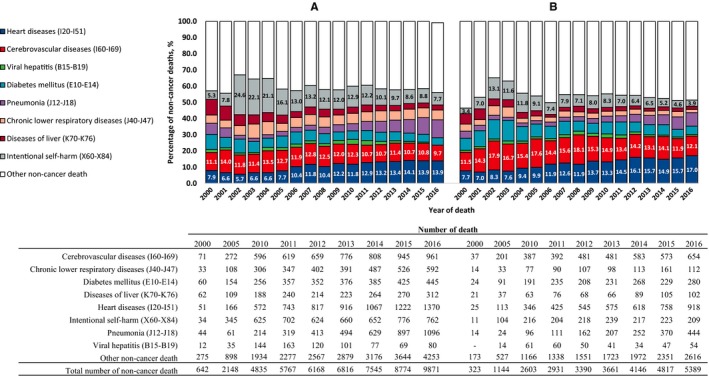
Proportion and number of major noncancer causes of death of (A) male and (B) female cancer patients with primary cancer diagnosed between 2000 and 2016. *Note.* Actual percentage of noncancer deaths for intentional self‐harm, heart diseases and cerebrovascular diseases are given in the figure as a highlight. ‐: number of death ≤10

From 2000 to 2016, the proportion of index cancer deaths among patients with cancer has gradually decreased, whereas the proportion of nonindex cancer deaths and noncancer‐related deaths has continuously increased (Figure 1), with noncancer‐related deaths notably increasing. In 2000, 965 patients with cancer died from noncancer‐related deaths, whereas 15 260 patients with cancer died from noncancer‐related deaths in 2016, which was 15.8‐fold higher than the number of noncancer‐related deaths in the year 2000 (Figure 1). The proportionate mortalities according to cause of death among total deaths are shown in Figure 2. This rapid increase in noncancer‐related deaths among patients with cancer was mainly due to the dramatic increase in deaths from cardiovascular diseases including heart and cerebrovascular disease (from 184 deaths in 2000 to 3903 deaths in 2016) and pneumonia (from 58 deaths in 2000 to 1540 deaths in 2016).

**Figure 3:**
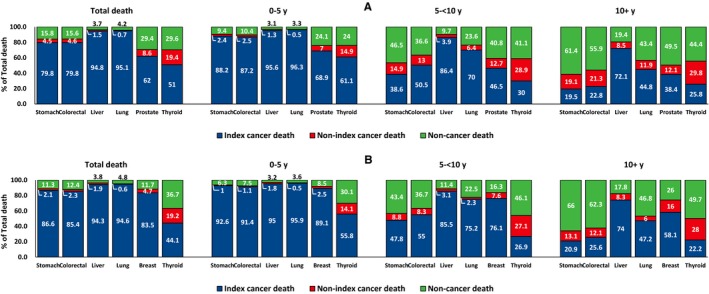
Proportionate mortality of (A) male and (B) female cancer patients with primary cancer of selected sites diagnosed between 2000 and 2016, stratified by time since cancer diagnoses. *Note.* 100% indicates the number of total deaths among cancer patients in each time period. The time of death was divided into <5 y after cancer diagnosis, from 5 to 10 y after cancer diagnosis and ≥10 y after cancer diagnosis

For individuals diagnosed with primary stomach, colorectal, liver, lung, or female breast cancer, most deaths within 5 years of diagnosis were due to the diagnosed cancer. However, the proportions of noncancer‐related deaths among total deaths were higher in patients with prostate (24.1%) and thyroid cancer (men, 24.0%; women, 30.1%) (Figure 3). Between 5 and 10 years after the diagnosis of cancer, 38.6%‐55.0% of the total deaths due to stomach, colorectal, and prostate cancers were due to the diagnosed cancer; furthermore, among those with stomach and colorectal cancers, <26% of overall deaths were attributed to the primary cancer after 10 years. In contrast, among patients with liver, lung and female breast cancer, 70%‐86% of the total deaths were due to the diagnosed cancer between 5 and 10 years after the diagnosis of cancer, and this proportion remained high even after 10 years, especially among the liver cancer patients (>70%).

### 3.3 Leading causes of noncancer‐related and nonindex cancer deaths

Among male patients with cancer, heart disease was the most common cause of noncancer‐related deaths, followed by cerebrovascular disease, intentional self‐harm, diseases of the liver, pneumonia, and chronic lower respiratory disease (Table 2). Among female patients with cancer, heart diseases were the most common cause of noncancer‐related deaths, followed by cerebrovascular disease, diabetes mellitus, intentional self‐harm, and pneumonia. These five common causes of death accounted for approximately 50% of all noncancer deaths among patients with cancer. Heart disease ranked higher among patients with cancer relative to the general population in both men and women. For male patients with cancer, pneumonia and tuberculosis received higher rankings relative to the general population, whereas female patients with cancer had a higher ranking in terms of liver disease relative to the general population.

**Table 2:** Leading causes of death among cancer patients compared to the Korean general population, 2000‐2016

Rank	Cancer patients			General population		
	COD	Count	(%^a^, nc%^b^)	COD	Count	(%^a^, nc%^b^)
Men						
1	Malignant neoplasms (C00‐C97)	633 273	(88.9, ‐)	Malignant neoplasms (C00‐C97)	740 183	(25.3, ‐)
2	Heart diseases (I20‐I51)	8 752	(1.2, 12.4)	Cerebrovascular diseases (I60‐I69)	239 129	(8.2, 10.9)
3	Cerebrovascular diseases (I60‐I69)	7 953	(1.1, 11.3)	Heart diseases (I20‐I51)	191 113	(6.5, 8.7)
4	Intentional self‐harm (X60‐X84)	7 710	(1.1, 10.9)	Intentional self‐harm (X60‐X84)	141 613	(4.8, 6.5)
5	Diseases of liver (K70‐K76)	5 500	(0.8, 7.8)	Diseases of liver (K70‐K76)	108 226	(3.7, 4.9)
6	Pneumonia (J12‐J18)	4 809	(0.7, 6.8)	Transport accidents (V01‐V99)	94 506	(3.2, 4.3)
7	Chronic lower respiratory diseases (J40‐J47)	4 358	(0.6, 6.2)	Diabetes mellitus (E10‐E14)	93 938	(3.2, 4.3)
8	Diabetes mellitus (E10‐E14)	3 948	(0.6, 5.6)	Chronic lower respiratory diseases (J40‐J47)	80 481	(2.7, 3.7)
9	Transport accidents (V01‐V99)	2 268	(0.3, 3.2)	Pneumonia (J12‐J18)	63 199	(2.2, 2.9)
10	Tuberculosis (A15‐A19)	1 250	(0.2, 1.8)	Falls (W00‐W19)	30 792	(1.1, 1.4)
Women						
1	Malignant neoplasms (C00‐C97)	351 874	(89.4, ‐)	Malignant neoplasms (C00‐C97)	433 657	(22.3, ‐)
2	Heart diseases (I20‐I51)	5 422	(1.4, 14.3)	Cerebrovascular diseases (I60‐I69)	259 402	(13.4, 17.2)
3	Cerebrovascular diseases (I60‐I69)	5 296	(1.3, 14.0)	Heart diseases (I20‐I51)	187 722	(9.7, 12.4)
4	Diabetes mellitus (E10‐E14)	2 494	(0.6, 6.6)	Diabetes mellitus (E10‐E14)	93 500	(4.8, 6.2)
5	Intentional self‐harm (X60‐X84)	2 433	(0.6, 6.4)	Intentional self‐harm (X60‐X84)	66 855	(3.4, 4.4)
6	Pneumonia (J12‐J18)	1 934	(0.5, 5.1)	Pneumonia (J12‐J18)	58 940	(3.0, 3.9)
7	Diseases of liver (K70‐K76)	1 667	(0.4, 4.4)	Hypertensive diseases (I10‐I13)	56 209	(2.9, 3.7)
8	Hypertensive diseases (I10‐I13)	1 170	(0.3, 3.1)	Chronic lower respiratory diseases (J40‐J47)	52 254	(2.7, 3.5)
9	Chronic lower respiratory diseases (J40‐J47)	1 135	(0.3, 3.0)	Transport accidents (V01‐V99)	34 395	(1.8, 2.3)
10	Transport accidents (V01‐V99)	669	(0.2, 1.8)	Diseases of liver (K70‐K76)	28 126	(1.4, 1.9)

Note

Causes of death were classified according to the 56 causes of death classification by the Statistics Korea. ICD‐10 codes for causes of death are in parentheses. Cancer patients were diagnosed from 2000 to 2016 and followed up to 31 December 2016. Causes of death of general population were ranked among people died from 2000 to 2016.

Abbreviations: COD, cause of death, nc, noncancer cause of death.

a Percentage of total number of deaths, which was calculated as the number of specific cause of death divided by the total number of death.

b Percentage of noncancer deaths, which was calculated as the number of specific cause of death divided by the total number of noncancer death.

Lung cancer was the most common nonindex cancer in patients with gastric, colorectal, thyroid, prostate, and female breast cancers, whereas gastric cancer was the most common nonindex cancer in patients diagnosed with lung cancer as the primary cancer (Table S1). Gallbladder and biliary tract cancers were the most common nonindex cancers only in patients with liver cancer as the primary cancer.

### 3.4 Risks of noncancer‐related deaths in patients with cancer relative to the general population

SMRs and AERs for common causes of noncancer‐related deaths in patients with cancer relative to the Korean general population are shown in Table 3. The mortality risks associated with intentional self‐harm were significantly higher in patients with cancer (SMR for men: 1.68 [95% CI: 1.63‐1.74]; SMR for women: 1.42 [95% CI: 1.33‐1.51]) relative to the South Korean general population. The AER for suicide death was 5.01 (95% CI: 4.48‐5.54) and 0.88 (95% CI: 0.65‐1.12) per 10,000 person‐years for male and female patients with cancer, respectively. When stratified according to cancer site, intentional self‐harm showed the highest SMRs for lung cancer (SMR for men: 2.99 [95% CI: 2.69‐3.30], SMR for women: 2.79 [95% CI: 2.08‐3.50]) (Table 4). The AERs for suicide death were also particularly high among patients with lung cancer (AER 16.01 [95% CI: 13.16‐18.85] and AER 4.52 [95% CI: 2.42‐6.61] per 10,000 person‐years, for men and women, respectively) (Table 4). In addition, the SMRs for intentional self‐harm were significantly higher in patients diagnosed with colorectal cancer (SMR for men: 1.40 [95% CI: 1.28‐1.52], SMR for women: 1.59 [95% CI: 1.32‐1.85]), stomach cancer (SMR for men: 1.68 [95% CI: 1.57‐1.79], SMR for women: 1.74 [95% CI: 1.46‐2.02]), and female breast cancer (SMR: 1.66 [95% CI: 1.44‐1.88]). In patients with cancer and liver disease, viral hepatitis was the most common cause of noncancer deaths and was associated with greater mortality risks compared to the general population (SMR for men: 46.71 [95% CI: 41.67‐51.75]; SMR for women: 56.44 [95% CI: 45.94‐66.94]). Although the mortality risks of heart disease in the Korean patients with cancer in our dataset were not higher than the South Korean general population (Table 2), heart disease was the most common cause of noncancer deaths in many patients with different cancer types including stomach, colorectal, lung, prostate, thyroid, and female breast cancers (Table 4).

**Table 3:** Standard mortality ratio for the leading noncancer causes of death among cancer patients compared to the Korean general population^a^

Rank	Men				Women			
	COD	Count	SMR [95% CI]^b^	AER [95% CI]^a^	COD	Count	SMR [95% CI]^b^	AER [95% CI]^a^
1	Heart diseases (I20‐I51)	4 427	0.90 [0.88 ,0.93]	−1.74 [−2.44, −1.03]	Heart diseases (I20‐I51)	2 290	0.96 [0.92, 1.00]	−0.24 [−0.62, 0.14]
2	Cerebrovascular diseases (I60‐I69)	3 440	0.73 [0.70, 0.75]	−4.80 [−5.46, −4.14]	Cerebrovascular diseases (I60‐I69)	2 053	0.83 [0.80, 0.87]	−1.18 [−1.55, −0.81]
3	Intentional self‐harm (X60‐X84)	3 300	1.68 [1.63, 1.74]	5.01 [4.48, 5.54]	Intentional self−harm (X60‐X84)	1 050	1.42 [1.33, 1.51]	0.88 [0.65, 1.12]
4	Pneumonia (J12‐J18)	2 648	0.95 [0.90, 1.00]	−0.23 [−0.59, 0.13]	Diabetes mellitus (E10‐E14)	967	0.83 [0.78, 0.88]	−0.56 [−0.82, −0.31]
5	Chronic lower respiratory diseases (J40‐J47)	1 846	0.97 [0.92, 1.01]	−0.24 [−0.68, 0.21]	Pneumonia (J12‐J18)	847	0.90 [0.81, 0.99]	−0.12 [−0.28, 0.03]

Note

Patients under 20 years old and over 85 years old at the midpoint of their death year were excluded. Causes of death were classified according to the 56 causes of death classification by the Statistics Korea. ICD‐10 codes for causes of death are in parentheses. Cancer patients diagnosed from 2000 to 2016 and followed up to 31 December 2016 and cause of death of cancer patients from 2012 to 2016.

Abbreviations: 95% CI, 95% Confidence Interval; AER, absolute excess risk; COD, cause of death; SMR, standard mortality ratio.

a Absolute excess risk(AER) was calculated as the difference between expected rate and observed rate per 10,000 person‐years.

b SMRs for the specific cause of death were calculated as the ratio of total observed deaths in cancer patients from 2012 to 2016 to the expected deaths in the general population from 2012 to 2016, respectively.

**Table 4:** Mortality risks of the leading noncancer causes of death by cancer sites in Korea

Cancer site	Men				Women			
	COD	Count	SMR [95% CI]^a^	AER [95% CI]^b^	COD	Count	SMR [95% CI]^a^	AER [95% CI]^b^
Stomach	Heart diseases (I20‐I51)^COD1^	1 196	0.92 [0.87, 0.98]	−1.45 [−2.87, −0.02]	Cerebrovascular diseases (I60‐I69)^COD1^	446	0.99 [0.90, 1.09]	−0.07 [−1.79, 1.65]
	Intentional self‐harm (X60‐X84)^SMR1^	864	1.68 [1.57, 1.79]	5.10 [4.04, 6.16]	Intentional self‐harm (X60‐X84)^SMR1^	149	1.74 [1.46, 2.02]	1.86 [0.98, 2.74]
Colorectal	Heart diseases (I20‐I51)^COD1^	735	0.74 [0.69, 0.80]	−4.98 [−6.58, −3.38]	Heart diseases (I20‐I51)^COD1^	372	0.80 [0.72, 0.89]	−2.72 [−4.41, −1.02]
	Intentional self‐harm (X60‐X84)^SMR1^	537	1.40 [1.28, 1.52]	3.01 [1.84, 4.18]	Intentional self‐harm (X60‐X84)^SMR1^	136	1.59 [1.32, 1.85]	1.51 [0.63, 2.38]
Liver	Viral hepatitis (B15‐B19)^COD1, SMR1^	330	46.71 [41.67, 51.75]	18.02 [16.01, 20.03]	Viral hepatitis (B15‐B19)^COD1, SMR1^	111	56.44 [45.94, 66.94]	18.78 [15.20, 22.37]
Lung	Heart diseases (I20‐I51)^COD1^	368	1.10 [0.99, 1.21]	2.20 [−1.22, 5.62]	Heart diseases (I20‐I51)^COD1^	113	1.03 [0.84, 1.22]	0.36 [−3.13, 3.85]
	Intentional self‐harm (X60‐X84)^SMR1^	365	2.99 [2.69, 3.30]	16.01 [13.16, 18.85]	Intentional self‐harm (X60‐X84)^SMR1^	59	2.79 [2.08, 3.50]	4.52 [2.42, 6.61]
Breast					Heart diseases (I20‐I51)^COD1^	246	0.91 [0.79, 1.02]	−0.35 [−0.97, 0.28]
					Intentional self‐harm (X60‐X84)^SMR1^	225	1.66 [1.44, 1.88]	1.25 [0.73, 1.77]
Prostate	Heart diseases (I20‐I51)^COD1^	369	0.69 [0.63, 0.75]	−9.54 [−12.41, −6.67]				
	Intentional self‐harm (X60‐X84)^SMR1^	252	1.10 [0.96, 1.23]	0.91 [−0.84, 2.67]				
Thyroid	Heart diseases (I20‐I51)^COD1, SMR1^	83	0.57 [0.45, 0.69]	−2.60 [−3.84, −1.36]	Heart diseases (I20‐I51)^COD1^	212	0.63 [0.54, 0.71]	−1.04 [−1.42, −0.66]
					Intentional self‐harm (X60‐X84)^SMR1^	163	0.73 [0.62, 0.84]	−0.50 [−0.82, −0.19]

Note

Patients under 20 y old and over 85 y old at the midpoint of their death year were excluded. Causes of death were classified according to the 56 causes of death classification by the Statistics Korea. ICD‐10 codes for causes of death are in parentheses. Cancer patients diagnosed from 2000 to 2016 and followed up to 31 December 2016 and cause of death of cancer patients from 2012 to 2016.

Abbreviations: 95% CI, 95% Confidence Interval; AER, absolute excess risk; COD, cause of death; SMR, standard mortality ratio.

COD1: Top ranked by the proportion of noncancer causes of death. SMR1: Top ranked by the SMR.

a SMRs for the specific cause of death were calculated as the ratio of total observed deaths in cancer patients from 2012 to 2016 to the expected deaths in the general population from 2012 to 2016, respectively.

b Absolute excess risk(AER) was calculated as the difference between expected rate and observed rate per 10 000 person‐years.

## 4 DISCUSSION

Cause of death statistics often serve as an important source of information concerning mortality patterns within a population and help to prioritize the allocation of health‐care resources. Despite the importance of this topic, a comprehensive cause of death landscape and noncancer mortality risks in patients with cancer remain to be determined.^4^, ^10^ In this study, we illustrated the patterns in cause of death statistics concerning patients with cancer relative to the general population utilizing a population‐based cancer registry data in South Korea linked to mortality. To our knowledge, this is the first study dealing with the cause of death evaluation in patients with cancer in South Korea based on nationally representative data. Therefore, our study findings address a previously unmet need for data regarding the causes of death and noncancer mortality risks in patients with cancer.

There have been very few comprehensive studies on causes of death reported in other countries. The changing cause of death patterns among patients with cancer reported in the United States showed similar trends to those of our findings; death patterns determined from the United States primary cancer data showed decreasing trends, whereas the proportion of noncancer deaths has increased in recent years.^4^ However, the magnitude of the change differs in South Korea where deaths from index cancer outnumber noncancer‐related deaths, whereas noncancer‐related deaths have started to surpass cancer deaths in some cancer cohorts in the United States.^4^

These findings may be partially due to differences in health‐care systems and treatment patterns between South Korea and the United States. In the United States and in South Korea, medical services are fee‐for‐service based.^19^ However, while South Korea has access to medical facilities for all insured citizens, many people in the United States rely on private health insurance, and there are significant differences in the types of medical services available depending on the type of employment.^20^ Despite these differences in health‐care systems, South Korea and the United States both report high survival rates in patients with cancer.^3^ Some differences in survival for specific types of cancer between the two countries have been reported, because of differences in stage distribution, national cancer screening systems, and treatment methods.^21^ However, the difference in the proportion of cancer deaths and noncancer‐related deaths between the South Korea and the United States may mainly be attributed to the difference in the major causes of death among the general population. As an example, death from cancer is much higher in South Korea than death from heart disease, whereas heart disease is the most common cause of death in the United States.^10^, ^22^ In 2015, the mortality rate for ischemic heart disease was 113 per 100 000 people in the United States compared to 38 per 100 000 people in South Korea, which was approximately one‐third that of the United States.^23^

Primary cancer remains the leading cause of death among Korean patients with cancer. In particular, >70% of deaths in patients with liver and lung cancers were due to the primary cancers between 5 and 10 years after the initial diagnosis. Even among female patients with breast cancer and among patients with prostate cancer, which are usually cancers with a good prognosis, the majority of deaths were attributed to the primary cancer, although the absolute number of deaths was relatively small. These findings suggest that physicians might consider extending the follow‐up period beyond 5 years after cancer diagnosis for some cancer cohorts. Additionally, a continued follow‐up might be needed for breast cancer survivors, consistent with many previous studies demonstrating continuous excessive mortality beyond 5 years after the diagnosis of breast cancer^24^, ^25^ and the relative commonality of late breast cancer recurrence.^26^ In contrast, deaths related to second primary cancers or other causes account for a relatively small proportion of overall deaths.

Noncancer‐related deaths have increased continuously from 2000 to 2016. The recent increase in noncancer‐related deaths is mainly attributable to an increase in deaths from cardiovascular disease and pneumonia among patients with cancer. In South Korea, mortality from cardiovascular disease and pneumonia has continuously increased among the general population also.^27^, ^28^ This phenomenon could be explained as due to a rapidly aging population with an extended life expectancy involving both patients with cancer and the general population.^27^ Further, cancer survivors are likely to experience competing risks of noncancer‐related death as they continue to survive. For example, noncancer‐related deaths have begun to surpass cancer deaths in some cancer cohorts in the United States that include patients expected to have long‐term survival.^4^

The leading noncancer‐related causes of death in patients with cancer were similar to the general population; however, patients with cancer faced a higher risk of mortality from suicide compared to the general population. In our study, patients with cancer had an approximately 1.4‐fold to 1.6‐fold higher risk of suicide mortality than the Korean general population. Similarly, a Swedish study reported higher risks of death from suicide among patients with cancer than among the general population.^29^ Several other studies have reported increased risk of suicide after cancer diagnosis including studies involving Asian patients.^30^, ^31^ These increased mortality risks might be attributable to psychosocial stress,^29^ and economic and family difficulties among patients with cancer.^32^, ^33^

In our study, the number of cardiovascular disease‐related deaths increased and the mortality risks for patients with cancer were lower relative to those of the Korean general population. As this study evaluated the underlying cause of death, treatment‐related cardiotoxicity was likely to have been reported as a cancer death rather than a noncancer‐related death. In addition, the follow‐up periods may have been insufficient for observing long‐term cardiotoxicity or death due to cardiovascular‐related comorbid conditions for the recently diagnosed patients with cancer, where competing risks of noncancer‐related death are expected.^34^ Conversely, the relatively lower cardiovascular mortality might be the result of a favorable improvement in lifestyle or behavioral changes in cancer survivors after cancer diagnosis,^35^ or may reflect the relatively low rate of ischemic cardiovascular disease in South Korea.^23^

Nonindex cancer accounted for a substantial proportion of deaths 5 years following the first primary cancer diagnosis. Lung cancer was the leading cause of nonindex cancer death in most patients with multiple primary cancers, even for patients with a prostate or thyroid tumor as the first primary diagnosis (Table S1). Patients with cancer of the thyroid were also at risk of secondary (nonindex) cancer‐related deaths, which additionally highlights the importance of continuous monitoring and follow‐up for cancer survivors in relation to their secondary cancer diagnosis and related mortality risk.^36^, ^37^ Our findings also suggest that patients diagnosed with nonindex cancer could be at a higher risk of death than patients with a single primary cancer. Indeed, among patients diagnosed with pediatric cancers, many previous studies have reported a higher risk of nonindex cancer‐related mortality.^38^, ^39^

Our findings address the previously unmet need for data regarding the causes of death and noncancer‐related mortality risks among patients with cancer compared to a general population, and provide useful information concerning the management of cancer survivors, as derived from a representative nationwide database. However, our findings should be interpreted with care as our study had some limitations. First, the reported causes of death may have included errors or misclassifications. However, we used the cause of death information from Statistics Korea, where official cause of death statistics is reported annually in South Korea.^10^ Statistics Korea has made ongoing efforts to improve accuracy in the cause of death data collection.^10^ Moreover, we applied the SEER cause‐specific death classification algorithm for the cause of death, as developed by the National Cancer Institute, United States,^13^, ^14^ to enhance the accuracy of cause of death classifications concerning patients with cancer. Second, when estimating the SMR for noncancer‐related causes of death according to cancer sites, the number of noncancer‐related deaths was small; therefore, the corresponding results should be viewed with caution. Given this factor, we focused mostly on the main causes of noncancer‐related deaths according to cancer sites. The SMRs were adjusted for sex and age and presented for descriptive purposes. Third, a direct comparison in terms of cause of death proportions between patients with cancer in the United States and in South Korea poses difficulties because the major types of cancer, stage distributions, and medical health‐care systems differ between the two countries. Causal interpretations in relation to diagnosed cancer and noncancer‐related causes of death should be applied cautiously, as cancer survivors may experience differing health conditions and related behaviors compared to the general population. Assessing factors associated with noncancer‐related health in cancer survivors relative to the general population was not the scope of this study but will be addressed in future research. Finally, this study focused mainly on mortality risk based on the time of death, and did not consider mortality risk variations with respect to the period of cancer diagnosis.

## 5 CONCLUSIONS

In summary, most patients with cancer in South Korea died from their primary cancer, unlike their United States counterparts. However, despite the predominance of index cancer deaths, the proportion of noncancer‐related deaths among all deaths increased over time. Our findings suggest that, even in terms of the standard 5‐year survival as a determinant of a cancer “cure,” some patients with cancer (eg, liver, lung, and female breast cancer) may require an extended follow‐up beyond this time point as the primary cancer remains the predominant cause of death. These changing noncancer mortality patterns suggest that a stronger focus is needed for noncancer‐related health issues in the care and management of cancer survivors. A rapid increase in deaths due to cardiovascular disease and higher mortality risks from suicide among patients with cancer highlight the need for preventing cardiovascular disease and suicide in cancer survivorship care.

## CONFLICT OF INTEREST

The authors have no conflict of interest to declare.

## AUTHOR CONTRIBUTIONS

Hyunsoon Cho, PhD made substantial contributions to the conception of the work, acquisition and interpretation of the data, and wrote the manuscript; Chang‐Mo Oh, MD, PhD wrote the manuscript, contributed to the study design and interpretation of the data; Dahhay Lee, MS conducted the data analysis and made the tables and figures; Hyun‐Joo Kong, MS, Kyu‐Won Jung, MS and Young‐Joo Won, PhD made substantial contributions to the acquisition of the data, critically revised the study protocol and manuscript; Seokmin Lee, MS made substantial contributions to the acquisition and interpretation of the data.

## Data Availability

Data that support the findings of this study can be requested from the Korea Central Cancer Registry (https://kccrsurvey.cancer.go.kr:10443/index.do) and the Statistics Korea (https://mdis.kostat.go.kr/index.do). The data are not publicly available due to privacy or ethical restrictions.
