# Supplementary material for: Causes of death among cancer patients in the era of cancer survivorship in Korea: Attention to the suicide and cardiovascular mortality
Source: Cancer Med. 2020 Jan 20;9(5):1741–52. doi: 10.1002/cam4.2813 (PMC7050076; doi:10.1002/cam4.2813)
Supplement: Supplementary file 1 [file CAM4-9-1741-s001.docx]

**Supplementary Table 1. Leading causes of non-index cancer death in cancer patients**^†^ **with multiple primaries in Korean, 2000-2016**

| **Cancer Site** | **Number of Death** | **COD** | **Count** | **%**^‡^ |
| --- | --- | --- | --- | --- |
| **Stomach** | 13,100 | Malignant neoplasm of trachea, bronchus and lung (C33-C34) | 2,218 | 16.9 |
| **Colorectal** | 12,481 | Malignant neoplasm of trachea, bronchus and lung (C33-C34) | 1,257 | 10.1 |
| **Liver** | 8,049 | Malignant neoplasm of gallbladder, other and unspecified parts of biliary tract (C23-C24) | 1,332 | 16.5 |
| **Lung** | 10,804 | Malignant neoplasm of stomach (C16) | 265 | 2.5 |
| **Breast^¶^** | 2,368 | Malignant neoplasm of trachea, bronchus and lung (C33-C34) | 173 | 7.3 |
| **Prostate** | 3,591 | Malignant neoplasm of trachea, bronchus and lung (C33-C34) | 702 | 19.5 |
| **Thyroid** | 2,201 | Malignant neoplasm of trachea, bronchus and lung (C33-C34) | 414 | 18.8 |
| *Note.* The cause of non-index death was classified according to the list of 24 cancer sites which was officially used in the Korean Central Cancer Registry. ICD-10 codes for causes of death are in parentheses.  COD=cause of death  † Cancer patients diagnosed from 2000 to 2016 and followed up to December 31, 2016 and cause of death of cancer patients from 2012 to 2016. The first of the multiple primary tumors were considered as an index-cancer.  ‡ Percentage of total death, which was calculated as the number of specific cause of death divided by total number of death of cancer patients with multiple primaries.  ¶ Female only. | | | | |
